# Evaluating the quality of educational TikTok videos on diabetic retinopathy: a cross-sectional study

**DOI:** 10.3389/fpubh.2025.1718587

**Published:** 2025-12-18

**Authors:** Ligang Jiang, Wencan Wu, Xin Jiang, Fangzheng Jiang

**Affiliations:** 1 Department of Ophthalmology, Quzhou Affiliated Hospital of Wenzhou Medical University, Quzhou People’s Hospital, Quzhou, Zhejiang, China; 2 The Eye Hospital, School of Ophthalmology & Optometry, Wenzhou Medical University, Wenzhou, China; 3 Oujiang Laboratory (Zhejiang Lab for Regenerative Medicine Vision and Brain Health), Wenzhou, Zhejiang, China; 4 Wenzhou Institute, University of Chinese Academy of Sciences, Wenzhou, China; 5 Quzhou College of Technology, Quzhou, Zhejiang, China

**Keywords:** diabetic retinopathy, health education, social media, TikTok, video quality

## Abstract

**Background:**

Diabetic retinopathy (DR) is a leading cause of vision loss among working-age adults, and enhancing public health literacy through effective education is crucial for its prevention and management. With the rise of mobile internet and short video platforms such as TikTok, new opportunities have emerged for disseminating medical knowledge. However, concerns remain regarding the accuracy and quality of this content.

**Methods:**

A cross-sectional search was conducted on September 18, 2025. A total of 200 Mandarin-language TikTok videos directly relevant to DR were included after screening. Baseline characteristics, uploader type, and user engagement metrics were extracted. Video quality was assessed independently by two trained reviewers using the DISCERN tool and the Patient Education Materials Assessment Tool for Audiovisual Materials (PEMAT-A/V). Content coverage was evaluated against the American Academy of Ophthalmology (AAO) Preferred Practice Pattern®. Inter-rater reliability was measured by intraclass correlation coefficients (ICCs). Group comparisons and correlation analyses were performed.

**Results:**

Significant differences were observed in quality scores across uploader categories (one-way ANOVA, *p* < 0.001). Non-profit organizations achieved the highest DISCERN scores (59.4 ± 8.2) and PEMAT-A/V understandability (88.5%), while for-profit accounts had the lowest DISCERN scores (23.0 ± 6.5; understandability 61.5%). Videos from non-profit sources also demonstrated balanced coverage across six core DR themes (14–20% per theme). Inter-rater reliability was excellent for all tools (ICC range 0.825–0.933). Engagement metrics were strongly correlated with DISCERN scores (likes *r* = 0.76, comments *r* = 0.64, favorites *r* = 0.73, shares *r* = 0.71; all *p* < 0.05), whereas video duration showed no significant correlation with quality (*p* > 0.05).

**Conclusion:**

The quality of DR-related educational short videos on TikTok varies widely, with the source of the video emerging as the key determinant. High-quality content from non-profit organizations and medical professional users not only demonstrates greater reliability but also fosters comprehensive health education. Strengthening professional participation, platform regulation, and evidence-informed communication strategies is essential to maximize the potential of short videos in DR health education and ultimately improve patient outcomes.

## 1 Introduction

Diabetic Retinopathy (DR), a principal microvascular complication of diabetes, represents the foremost cause of irreversible blindness in working-age adults globally (1, 2). As the global prevalence of diabetes continues to climb, the incidence and morbidity of DR have correspondingly increased, creating a substantial public health and socioeconomic burden (3–5). Nevertheless, the progression of DR can be significantly delayed or prevented through early screening, prompt intervention, and tight control of both glycemic and blood pressure levels (6–8). Therefore, it is imperative that both patients and the general public possess adequate awareness and health literacy concerning DR. Consequently, the provision of high-quality, comprehensible, and readily accessible public health education is of paramount importance for the prevention and control of DR.

With the recent and rapid advancement of mobile internet technologies, short videos, such as those on YouTube and TikTok have emerged as the most rapidly expanding medium for information dissemination (9–13). short videos hold prominent advantages over traditional formats such as text with images or long-form videos, characterized by their rapid dissemination, extensive reach, bite-sized content, and engaging visual presentation. This medium has substantially reduced the threshold for obtaining health information (14, 15), establishing itself as a crucial new avenue for the public to gain medical knowledge on topics including DR (16–18). Moreover, physicians and healthcare organizations have progressively leveraged these platforms for science popularization to enhance public health consciousness (19).

Despite the great potential of short videos in education (20), the quality, accuracy, and reliability of their content face severe challenges (10, 21). Due to the lack of strict content review mechanisms, these platforms are inundated with a large volume of short videos produced by non-professionals or for commercial marketing purposes (22). This content may contain misleading information, inaccurate diagnostic and treatment advice, or even erroneous health concepts (23–25), which, for DR patients and their families who need to follow long-term and precise management plans, could lead to anxiety, delayed medical consultation, or the adoption of improper treatments, ultimately endangering their vision.

However, to date, there have been no systematic and objective quality assessment studies on health education content about the specific and important disease of DR on mainstream short video platforms. This study aims to use a cross-sectional design to systematically retrieve and evaluate the information quality and reliability of education short videos about DR on current mainstream short video platforms.

## 2 Materials and methods

### 2.1 Search strategy and video selection

**Figure 1:**
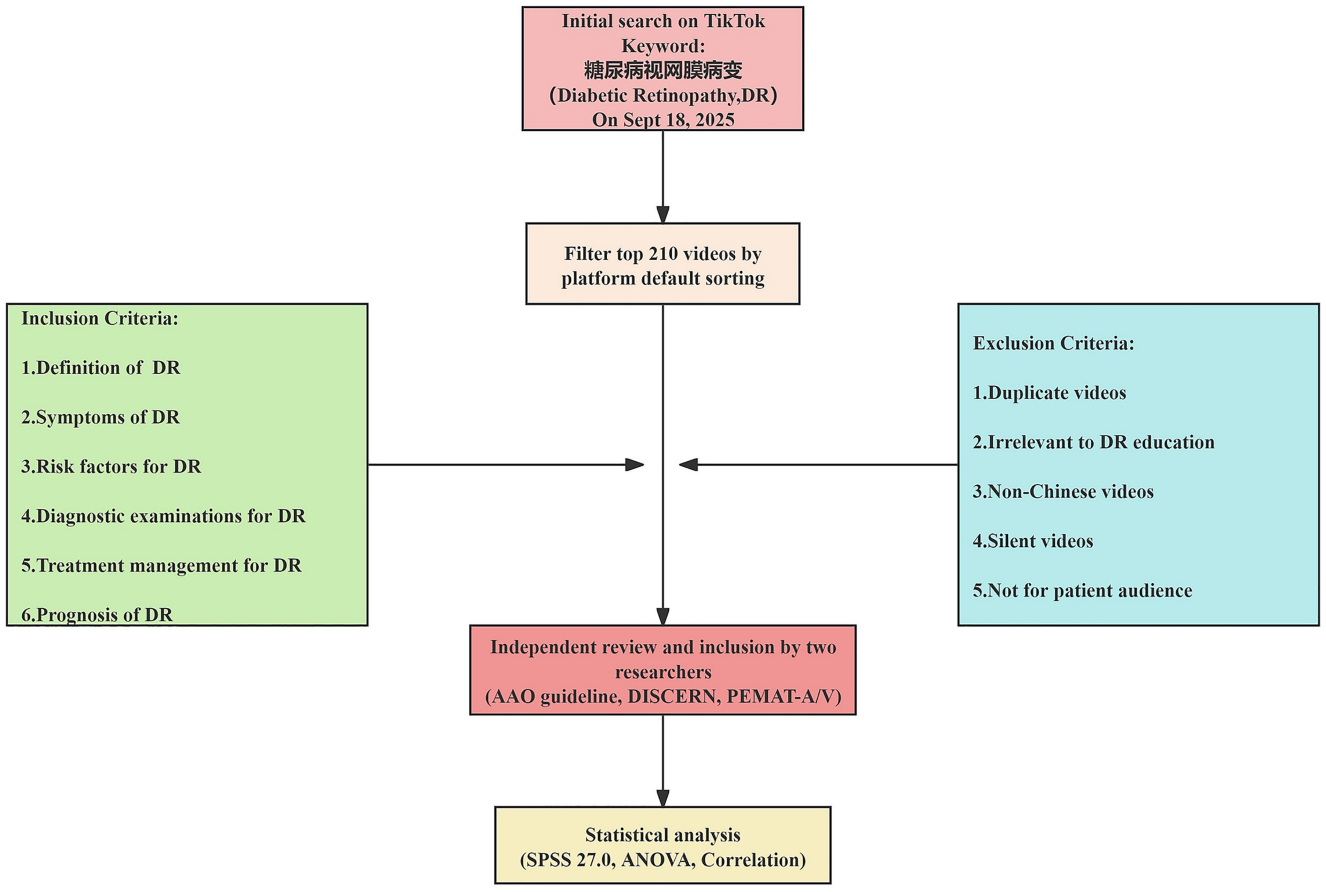
Study flow diagram.

On September 18, 2025, to minimize interference from personalized recommendation algorithms, we cleared the browser history and cookies before the search. We entered the keyword “diabetic retinopathy” (糖尿病视网膜病变) into TikTok (the mainland China version). In accordance with previous research practices and to ensure a representative sample, the top 210 videos sorted by the platform’s default ranking were selected for screening. The resulting videos were then assessed for eligibility against predefined inclusion criteria by two researchers, with those directly relevant to DR being included for final analysis (Figure 1).

- Inclusion criteria: Videos were included if they described any of the following aspects of DR: definition, classification, symptoms, risk factors, diagnostic, treatment, management, or prognosis.
- Exclusion criteria: Videos were excluded if they were duplicates, unrelated to DR education, not in Mandarin Chinese, silent, or not targeted at a patient audience.
- Screening process: Two researchers independently assessed each video for eligibility. Any disagreements were resolved through discussion or, if necessary, by a third researcher to reach a consensus.

### 2.2 Baseline data extraction

Two trained researchers independently and systematically extracted baseline characteristics from each included video and recorded the data in a pre-designed, structured spreadsheet. Extracted variables included the unique video identifier (URL), upload date, uploader source, video duration, account registration country and region, and video title. User engagement metrics were also recorded, including the number of likes, comments, favorites, and shares. All information was manually extracted from publicly available TikTok webpages. Only aggregated, non-identifiable data (e.g., counts of likes, comments, favorites, and shares) were recorded; no personal identifiers or user-level data were collected or stored. The study procedures were conducted in compliance with TikTok’s Terms of Service and Privacy Policy, and the analysis was limited to publicly accessible, de-identified data.

Based on the uploader’s account name and verification status, videos were categorized into four groups: Medical professional users (ophthalmologists or other healthcare professionals). Non-medical users (general public and patients). For-profit organizations (e.g., corporations, commercial media). Non-profit organizations (e.g., healthcare institutions, news agencies). This classification was based on the account’s verification information and profile description.

### 2.3 Video assessment

To ensure the scientific validity of the evaluation, this study used the *Diabetic Retinopathy Preferred Practice Pattern®* published by the American Academy of Ophthalmology (AAO) as the standard for content assessment (26). The videos were analyzed based on two dimensions: information coverage and quality. Two researchers with backgrounds in DR research first jointly reviewed the top 10 ranked videos to familiarize themselves with the framework and standardize their understanding, after which they independently conducted the formal assessment for all included videos. Content completeness was assessed using a framework adapted from Goobie et al. (27) for evaluating educational videos, which covered six core aspects: DR definition, DR classification, DR symptoms, DR risk factors, DR diagnostic methods, DR treatment and management.

Video quality was assessed using two validated tools: The DISCERN tool (16 items, divided into reliability, treatment information, and overall quality) (28, 29); The Patient Education Materials Assessment Tool for Audiovisual Materials (PEMAT-A/V) (17 items, divided into understandability and actionability), which was scored as “agree,” “disagree,” or “not applicable” (11, 30). Two assessors independently scored all videos. Before scoring, they received standardized training and referred to the latest AAO guidelines to ensure consistency. The average of the two assessors’ scores was used for the final statistical analysis, and the intraclass correlation coefficient (ICC) was calculated.

### 2.4 Statistical analysis

Statistical analyses were performed using IBM SPSS Statistics for Windows, Version 27.0 (IBM Corp., Armonk, NY, USA). The Shapiro–Wilk test was used to evaluate the normality of continuous variables. Normally distributed continuous variables were expressed as mean ± standard deviation (SD), whereas non-normally distributed variables were presented as median and interquartile range (IQR). Categorical data are presented as counts and percentages (*n*, %). Inter-rater reliability between the two reviewers was evaluated using the ICC. Differences in quality scores across uploader categories were first analyzed using ANOVA. When the assumption of homogeneity of variances was violated, Welch’s ANOVA or the Kruskal–Wallis test was applied, as appropriate. Whenever an omnibus test was statistically significant, pairwise *post hoc* comparisons between uploader groups were conducted using Bonferroni-adjusted significance levels to control for type I error (for parametric tests) or Dunn’s post hoc test with Bonferroni correction (for non-parametric data). Pearson’s correlation coefficient was used to assess the relationship between video characteristics and quality scores for normally distributed variables, whereas Spearman’s rank correlation coefficient was used for non-normally distributed variables. A two-tailed *p* value < 0.05 after adjustment for multiple comparisons was considered statistically significant for all analyses.

## 3 Results

### 3.1 Analysis of basic video parameters

A total of 210 TikTok videos were initially identified using the keyword “diabetic retinopathy.” After excluding 10 videos that did not meet the inclusion criteria (4 duplicates, 2 videos unrelated to DR education, 3 non-Mandarin videos, and 1 silent video without narration), 200 DR-related educational short videos were included in the final analysis. All included videos were verified by the platform. The majority of these videos were uploaded by medical users (174/200, 87.0%), followed by non-profit organizations (10/200, 5.0%). Non-medical users and for-profit organizations each accounted for the fewest videos (8/200, 4.0% each). Across all videos, the median numbers of likes, comments, favorites, and shares were 274.0, 15.0, 87.0, and 52.0, respectively, and the median duration was 70.5 s. Overall, there were significant differences in user engagement based on the uploader type, with videos from non-profit organizations and medical users showing higher engagement metrics (see Table 1 for details).

**Table 1:** Basic parameters of videos.

Video parameters	Individual user		Organizational user		Overall
	Medical (*n* = 174)	Non-medical (*n* = 8)	For-profit (*n* = 8)	Non-profit (*n* = 10)	
Likes	304.50 (106.25, 855.00)	18.50 (14.25, 31.50)	17.00 ± 6.48	18,000.00 (10,680.25, 59,250.00)	274.00 (87.25, 906.50)
Comments	16.00 (8, 50.25.00)	3.5 (1.25, 12.00)	1.38 ± 0.92	633.00 (345.75, 3,882.50)	15.00 (7.00, 54.75)
Favorites	100.00 (36.00, 258.25)	5.75 ± 4.30	2.75 ± 2.32	5,292.50 (3,135.00, 17,000.00)	87.00 (26.25, 300.25)
Shares	57.50 (16.75, 213.00)	3.50 ± 2.83	4.75 ± 3.85	6,215.50 (1,726.00, 29,000.00)	52.00 (12.25, 232.75)
Times	71.00 (47.00, 123.00)	80.25 ± 35.78	48.25 ± 15.92	121.50 (51.50, 218.00)	70.50 (47.00, 122.50)

### 3.2 Video quality analysis

**Figure 2:**
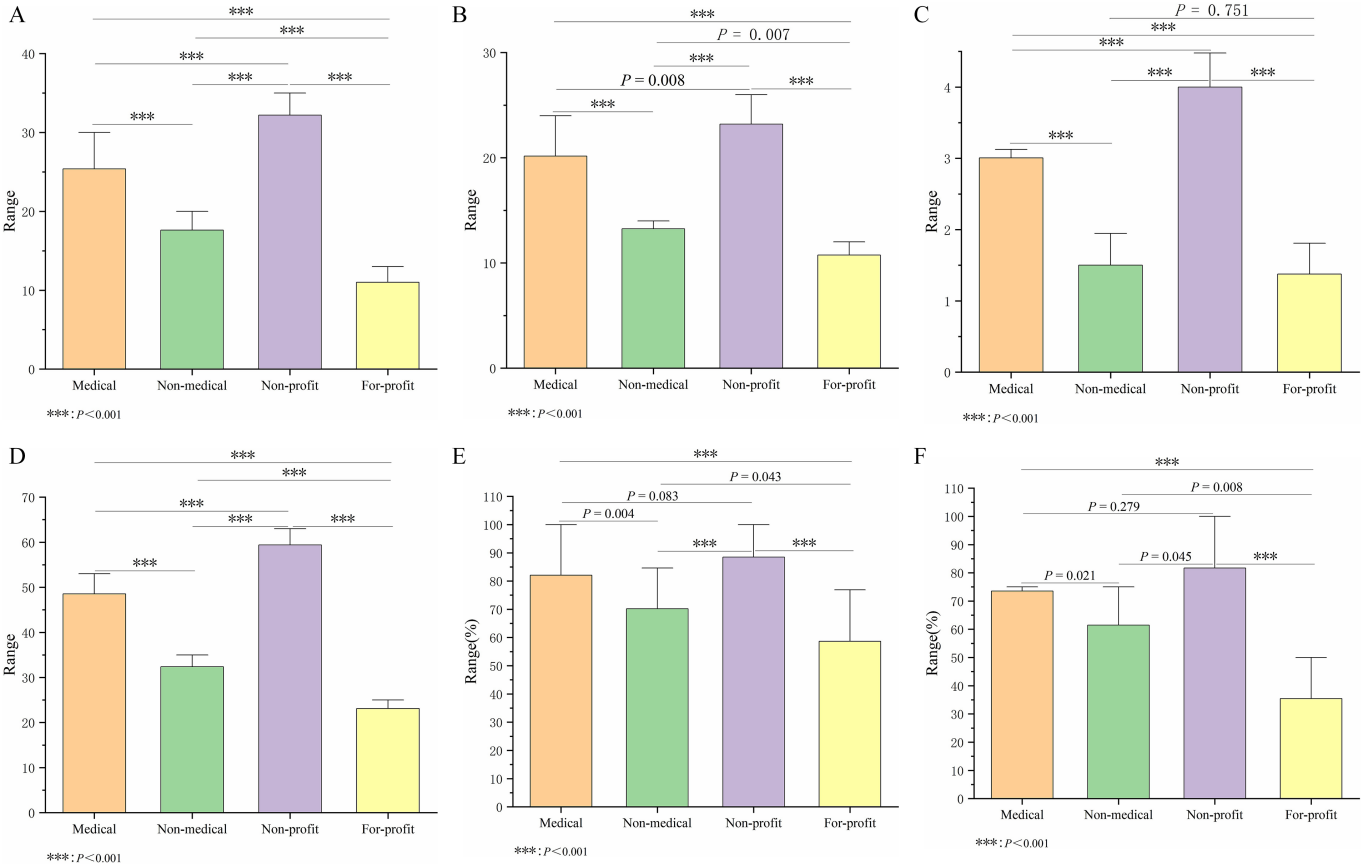
Intergroup comparison among four different video source users. **(A)** Reliability score. **(B)** Treatment choice score. **(C)** Overall quality score. **(D)** DISCERN tool score. **(E)** Understandability score. **(F)** Actionability score.

The inter-rater agreement was high for video reliability (ICC = 0.886), treatment choice (ICC = 0.933), DISCERN tool scores (ICC = 0.871), overall quality score (ICC = 0.879), understandability (ICC = 0.839), and actionability (ICC = 0.825) (all *p* < 0.001). In the subgroup analysis, which assessed video quality by uploader source, the results showed significant differences among user types across all quality assessment dimensions (*p* < 0.001) (Table 2). For video reliability, DISCERN tool scores, and overall quality score, videos from non-profit organizations scored the highest (32.20, 59.40, and 4.00, respectively), followed by those from medical users (25.00, 48.00, and 3.00, respectively). Videos from for-profit organizations had the lowest scores on these metrics (12.00, 23.00, and 1.00, respectively), See Table 2 and Figures 2A–D for details. A similar trend was observed in the assessment using the PEMAT-A/V tool. Videos from non-profit organizations had the highest understandability scores (88.46%), closely followed by those from medical users (84.62%). In terms of actionability, videos from medical users and non-profit organizations tied for the highest score (both 75.00%), whereas videos from for-profit organizations scored significantly lower (33.33%) (Table 2 and Figures 2E,F).

**Table 2:** Scores by different video sources.

Variables	Individual users		Organization users		*p* Value
	Medical (*n* = 174)	Non-medical (*n* = 8)	For-profit (*n* = 8)	Non-profit (*n* = 10)	
Videos reliability (*n* = 200)	25.00 (24.00, 27.00)	17.63 ± 1.77	12.00 ± 0.71	32.20 ± 2.04	<0.001
Treatment choice (*n* = 200)	20.00 (19.00, 21.00)	13.00 (13.00, 13.75)	11.40 ± 0.89	23.20 ± 2.20	<0.001
DISCERN tool scores (*n* = 200)	48.00 (47.00, 50.00)	32.38 ± 2.13	23.00 (21.50, 25.00)	59.40 ± 1.90	<0.001
Overall quality score (*n* = 200)	3.00 (2.00, 4.00)	1.50 (1.00, 2.00)	1.00 (1.00, 2.00)	4.00 ± 0.67	<0.001
PEMAT-A/V understandability (%)	84.62 (76.92, 92.31)	70.19 ± 12.63	58.65 ± 16.41	88.46 ± 6.54	<0.001
PEMAT-A/V actionability (%)	75.00 (66.67, 75.00)	61.46 ± 15.39	33.33 (27.08, 45.83)	75.00 (75.00, 100.00)	<0.001

**Figure 3:**
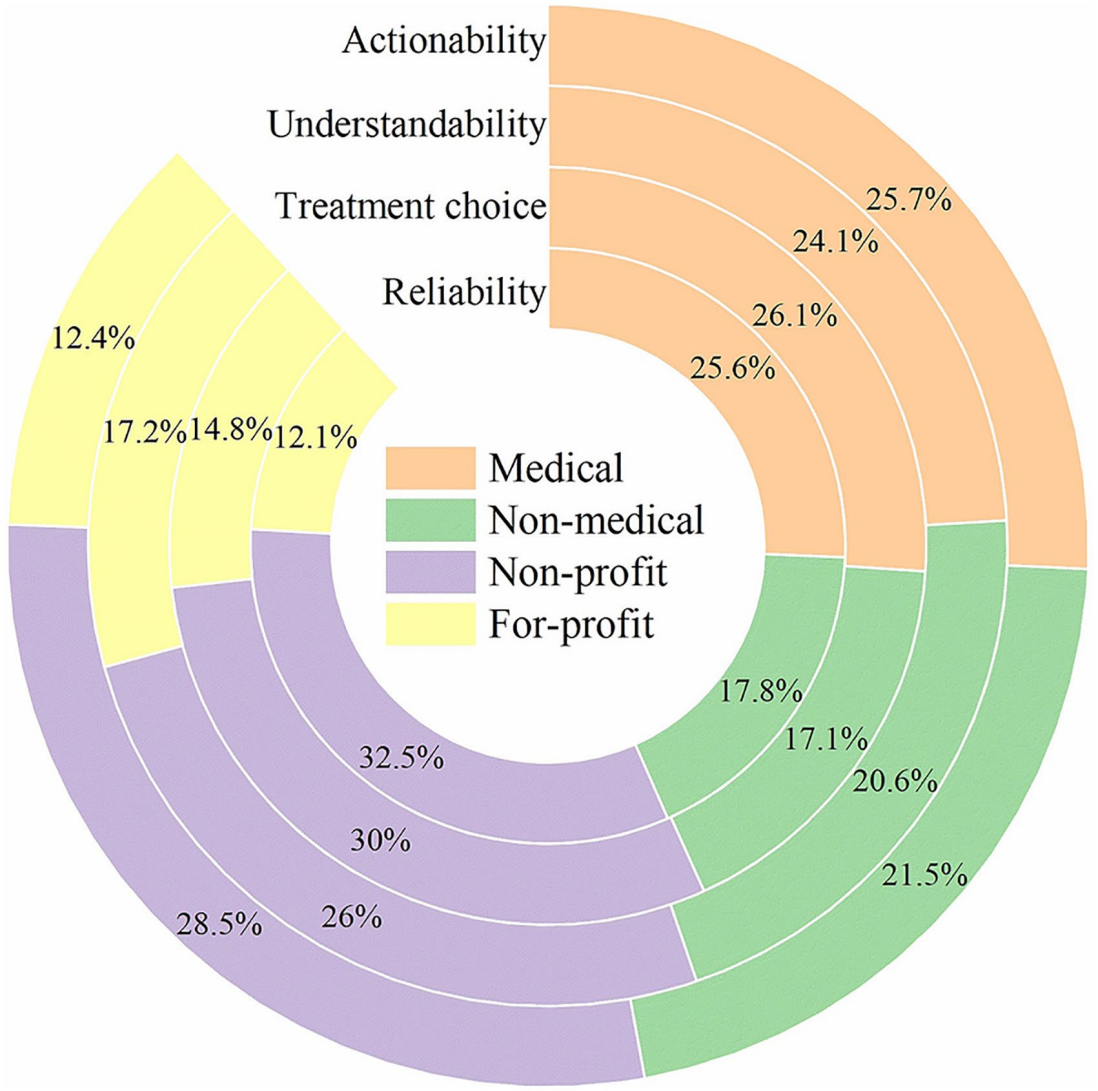
Comparison of the quality of the videos from four sources.

As shown in Figure 3, clear differences were observed in the proportional distribution of scores across uploader groups. Regarding Reliability, the Non-profit and Medical groups accounted for 32.5 and 25.6% of the total, respectively, whereas the Non-medical and For-profit groups contributed only 17.8 and 12.1%. For Treatment choice, the Non-profit and Medical groups contributed 30.0 and 26.1%, compared with only 17.1 and 14.8% for the Non-medical and For-profit groups. For Understandability, the Non-profit and Medical groups accounted for 26.0 and 24.1%, whereas the Non-medical and For-profit groups accounted for 20.6 and 17.2%. For Actionability, the Non-profit and Medical groups contributed 28.5 and 25.7%, while the Non-medical and For-profit groups contributed only 21.5 and 12.4%. Across all four dimensions, Non-profit and Medical uploaders consistently accounted for a larger proportion of the quality scores, whereas Non-medical and For-profit uploaders lagged behind.

### 3.3 Video content analysis

**Figure 4:**
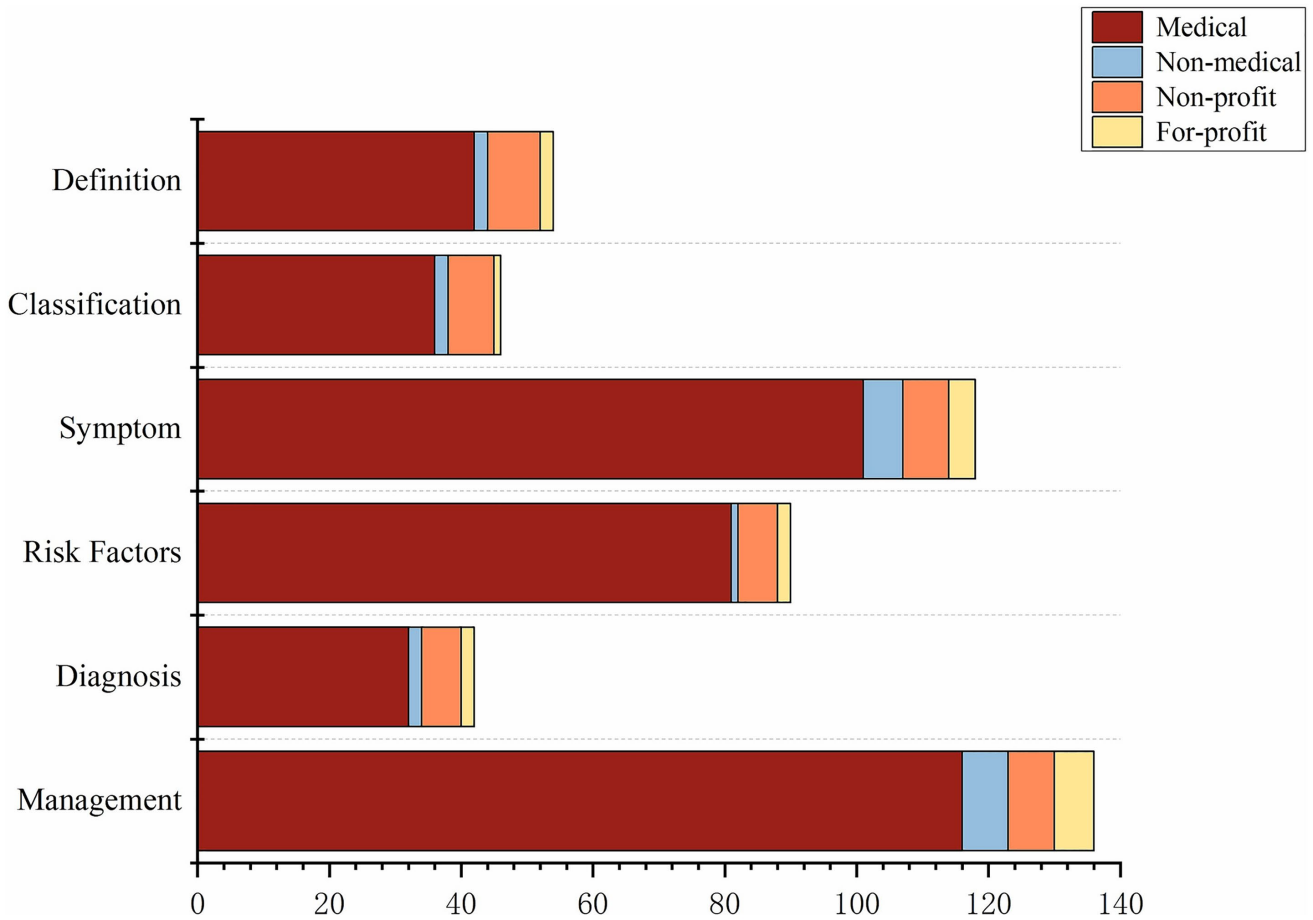
Videos from different sources cover various aspects of DR.

There were significant differences in the content theme distribution strategies among the different video sources. Specifically, the content strategies of medical professional users, non-medical users, and for-profit organizations were similar, with all three groups tending to prioritize practical topics. In content from medical professional users, “Management” (28.4%) and “Symptom” (24.8%) were the main components, whereas content from non-medical and for-profit sources was more focused, with the “Management” theme accounting for over 35% for both groups. In contrast, content from non-profit sources had the most balanced distribution, with coverage rates for the six themes all falling between 14 and 20%, indicating a more comprehensive educational strategy. In summary, videos from medical professional users, non-medical users, and for-profit organizations prioritized practical topics like “Management” and “Symptom,” while content from non-profit sources was more comprehensive and balanced across all basic knowledge themes (Figure 4).

### 3.4 Correlation analysis

**Figure 5:**
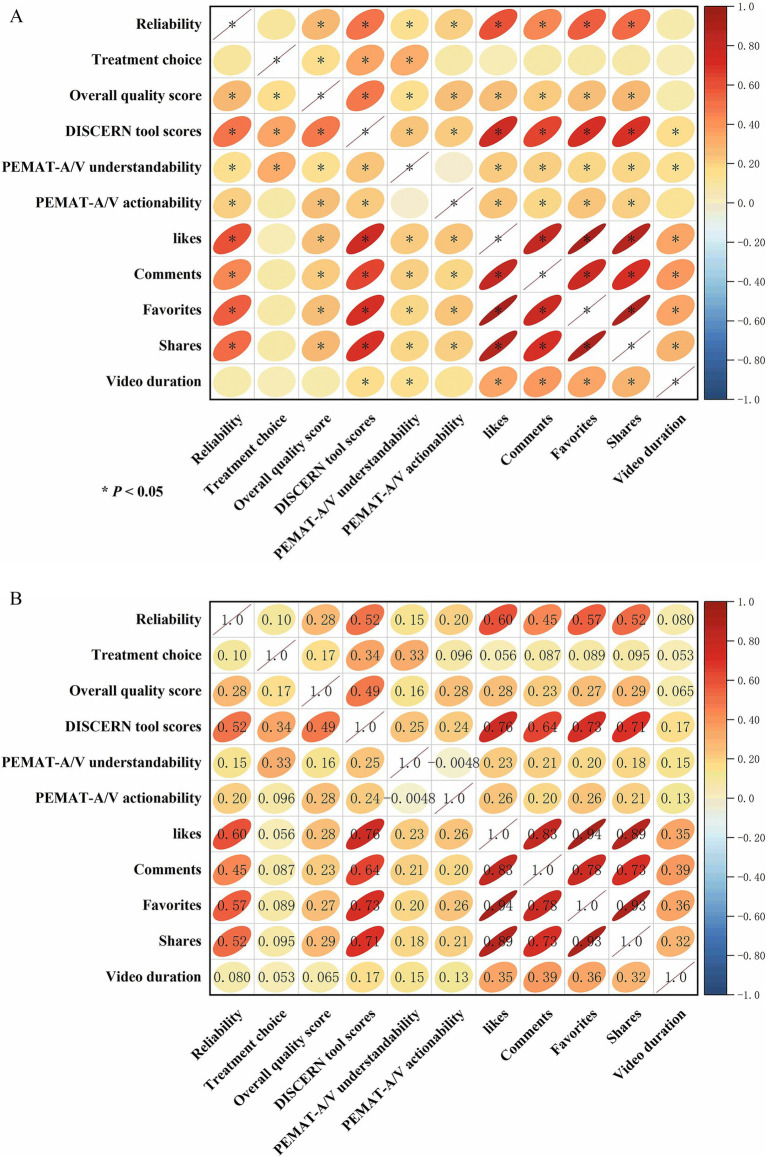
Correlation matrix illustrating the relationships between video quality assessment scores and user engagement metrics. **(A)** Visualization of the correlation coefficients. The color intensity and the shape of the ellipses represent the strength of the correlation; red indicates a positive correlation. Asterisks (*) denote a statistically significant correlation (*p* < 0.05). **(B)** Detailed matrix showing the specific correlation coefficients (*r*-values) in the upper triangle and the corresponding visual representation in the lower triangle.

Correlation analysis revealed significant positive correlations among the engagement metrics for DR-related videos on TikTok. Specifically, the number of likes was strongly and positively correlated with the number of comments (*r* = 0.83, *p* < 0.05), favorites (*r* = 0.94, *p* < 0.05), and shares (*r* = 0.89, *p* < 0.05). video duration showed a weak positive correlation with all engagement metrics (*r* = 0.32–0.39, *p* < 0.05). Among the medical information quality metrics, video reliability was moderately and positively correlated with the number of likes (*r* = 0.60, *p* < 0.05), comments (*r* = 0.45, *p* < 0.05), favorites (*r* = 0.57, *p* < 0.05), and shares (*r* = 0.52, *p* < 0.05), but it showed no significant correlation with video duration. The overall quality score, understandability, and actionability were all weakly and positively correlated with video engagement metrics (*p* < 0.05). The DISCERN tool score was strongly and positively correlated with the number of likes (*r* = 0.76, *p* < 0.05), comments (*r* = 0.64, *p* < 0.05), favorites (*r* = 0.73, *p* < 0.05), and shares (*r* = 0.71, *p* < 0.05), but had no significant correlation with video duration (Figure 5).

## 4 Discussion

To the best of our knowledge, this is the first study to systematically assess the quality and reliability of educational short video content concerning DR on the TikTok platform. The results indicate that although DR-related educational short videos are active in terms of user engagement and dissemination, their overall quality varies significantly. Notably, videos published by for-profit accounts generally had low information accuracy and educational value. In contrast, videos from non-profit organizations and medical professional users scored higher in terms of reliability, understandability, and actionability, demonstrating greater potential for health education.

Kong et al. (31) noted in their study of diabetes-related videos on TikTok that videos uploaded by non-profit organizations performed best on the DISCERN score, while those from commercial organizations scored the lowest. Analogously, the analysis by Wu et al. (32) of videos concerning hypertension and diabetes treatment on WeChat and TikTok revealed that the majority of videos were of low quality and did not satisfy established standards, indicating a pressing need to enhance the scientific rigor of medical information, even on platforms with vast numbers of users. The existing literature widely agrees that social media and short video platforms have great potential for disseminating medical and health information (33), but the quality of their content is inconsistent. In particular, videos produced by non-medical users or commercial accounts often contain one-sided or even erroneous information that may mislead patients (34). A similar trend was observed in a study on educational short videos about dry eye disease (35): although the videos had high understandability, their actionability was insufficient, and the coverage of basic knowledge was incomplete.

The findings of the present study align with the conclusions of previous research. We observed that videos concerning DR that were uploaded by non-profit organizations were superior to those from other sources in several aspects; they not only received the highest scores on both the DISCERN and PEMAT-A/V tools but also provided more thorough content coverage. In contrast to content from medical or commercial sources that frequently concentrated on pragmatic subjects like “management” and “symptoms,” non-profit organizations showed a greater tendency to systematically address the six crucial themes of DR, including its definition, classification, symptoms, risk factors, diagnosis, and management. Such a holistic and well-rounded educational approach is capable of balancing scientific rigor with effective dissemination, which aids in building a complete conceptual understanding of the disease for patients and consequently holds greater value in enhancing health literacy.

This study further revealed a moderate-to-strong positive correlation between user engagement indicators (e.g., likes, comments, favorites, shares) and information quality scores, which indicates that higher-quality content is more readily endorsed and shared by users (36). This result implies that the audience has some capacity for discernment; however, the extent of a video’s dissemination is likely still heavily influenced by its visibility and the platform’s algorithms (37). It is noteworthy that video duration were not significantly correlated with quality, indicating that the scientific rigor and the method of presentation are more crucial factors than duration alone. Consequently, for both platform promotion efforts and the health education practices of physicians, it is essential to focus on organically combining scientifically rigorous content with effective communication strategies (38, 39).

Notably, DR is a chronic, progressive microvascular complication of diabetes that requires life-long management and, in many cases, multidisciplinary care (40). Because early and even sight-threatening stages may be asymptomatic, patients are often unaware of the need for regular ocular screening and tight systemic control until irreversible damage has occurred (41). High-quality DR-related educational short videos should therefore do more than simply list symptoms or provide brief “management tips.” They should clearly explain what DR is and how it progresses, highlight key systemic and ocular risk factors, emphasize the importance of regular dilated fundus examinations and timely referral, and offer balanced information on evidence-based treatment options such as laser photocoagulation, intravitreal anti-VEGF therapy, and vitrectomy, together with realistic expectations regarding visual outcomes and the need for long-term follow-up (42, 43). In our sample, however, videos from medical, non-medical, and for-profit users tended to prioritize practical themes such as “management” and “symptoms,” whereas risk-factor education, screening recommendations, and long-term prognosis were relatively underrepresented (44). This pattern suggests substantial room for improvement in the completeness of DR-related health education on TikTok. From a communication perspective, it is equally important how these core messages are presented. Effective DR popular-science videos should use plain, patient-centered language, avoid excessive jargon, and employ intuitive visual aids—such as simple diagrams, fundus photographs, or animations—to illustrate retinal damage and treatment procedures in a way that is accessible to viewers with varying levels of health literacy. Information can be structured in a clear sequence, end with a specific call-to-action encouraging people with diabetes to undergo regular eye examinations, and explicitly remind viewers that online content cannot replace professional medical consultation. At the same time, creators should refrain from sensational titles, exaggerated promises of “cure,” or promotion of unproven remedies (45, 46), and should transparently disclose any commercial intent or sponsorship. Taken together, our findings imply that partnering with non-profit organizations and professional bodies to produce and promote videos that meet these content and presentation criteria may be a practical strategy to improve the quality and public-health impact of DR-related educational short videos on TikTok (47).

The present study is subject to certain limitations. First, our analysis was restricted to Mandarin-language DR videos on TikTok and relied on a single disease-specific keyword (Diabetic Retinopathy) for video retrieval. Although this term is the standard clinical designation used in Chinese guidelines and patient-education materials, this keyword-based strategy may have failed to capture videos that discussed DR using alternative lay expressions or more generic phrases related to diabetic eye disease. Moreover, TikTok is currently one of the most widely used short-video platforms in mainland China but represents only a portion of the broader Chinese short-video ecosystem; other popular platforms, such as Xiaohongshu and WeChat video channels, also host DR-related educational content, and the quality, framing, and user reach of the videos posted there may differ from those observed on TikTok. Our findings should therefore be interpreted as platform- and keyword-specific and may not be directly generalizable to other platforms or non-Mandarin language settings. Second, the cross-sectional design of this study captures video quality at only one specific time point. Given the extremely fast pace of content renewal and algorithm-driven content distribution on short-video platforms, both the visibility and composition of DR-related videos may change rapidly over time, and the applicability of our results is consequently constrained by their temporal nature. Furthermore, while internationally validated instruments such as DISCERN and PEMAT-A/V were employed to evaluate video quality, the effectiveness of health communication is also shaped by numerous audience-related factors, including educational attainment, media literacy, and cultural context. These audience-level variables were not incorporated into the present analysis and may modify how information of similar “objective” quality is actually perceived and acted upon. Subsequent studies could integrate user surveys and experimental designs to assess how various demographic groups comprehend, trust, and are behaviorally influenced by health-related short videos. Such work would provide a more nuanced and robust evidence base for developing an evidence-informed framework to guide the design and dissemination of DR-related public health communication on short-video platforms.

## 5 Conclusion

In conclusion, this study demonstrates that there is considerable variation in the overall quality of educational short videos about DR on the TikTok platform, and the source of the video is a key factor influencing its quality. Videos produced by non-profit organizations and medical professional users are of significantly higher quality than those from for-profit accounts and general users. In the future, promoting the standardized and scientific application of short videos in DR health education will require enhancing the participation of professionals, improving platform content review and regulatory mechanisms, and optimizing video content formats and presentation strategies, combined with long-term follow-up and effectiveness evaluation studies, to better improve patient health literacy and benefit public health.

## Data Availability

The original contributions presented in the study are included in the article/supplementary material, further inquiries can be directed to the corresponding author.
